# Mothers’ Health and Relationships With Adult Children: The Moderating Role of Gender and Race

**DOI:** 10.1093/geroni/igaa057.1670

**Published:** 2020-12-16

**Authors:** Catherine Stepniak, J Jill Suitor, Megan Gilligan

**Affiliations:** 1 Purdue University, West Lafayette, Indiana, United States; 2 Iowa State University, Ames, Iowa, United States

## Abstract

Consistent with theories of the life course and intergenerational solidarity, families are generally closely tied groups in which one family member’s event affects other members as well. Although the literature has documented that parents and adult children affect one another’s well-being, less is known about how parents’ health shapes relationship quality between family members. In this paper, we utilize data from the Within-Family Difference Study (WFDS) II to explore how mothers’ functional limitations affect relationship quality between mothers and their adult children, as reported by both family members. We hypothesized that the association between mothers’ health and intergenerational relationship quality would be moderated by gender and race. Using multi-level regression modeling, we found that mothers’ reports of relationship quality were not predicted by the presence of mothers’ functional limitations, nor were there any moderating effects of race or gender. In contrast, adult children who perceived that their mothers had limitations reported higher tension with them. Further, daughters were more likely than sons to report greater tension when they perceived that their mothers had limitations (differences between coefficients p < .10). White adult children reported lower levels of closeness and higher levels of tension when they perceived that their mothers had health limitations (differences between coefficients p < .05; p < .10 respectively). However, limitations did not predict Black children’s reports of closeness or conflict with mothers. This study sheds new light on the complex ways in which race and gender moderate the role of mothers’ limitations in intergenerational relationship quality.

